# 1875. Continued demographic shifts in hospitalised COVID-19 patients from migrant workers to a vulnerable and more elderly local population at risk of severe disease

**DOI:** 10.1093/ofid/ofac492.1502

**Published:** 2022-12-15

**Authors:** Jinghao Nicholas Ngiam, Srishti Chhabra, Wilson Goh, Meng Ying Sim, Tze Sian Liong, Nicholas W S Chew, Ching Hui Sia, Gail Brenda Cross, Paul Anantharajah Tambyah

**Affiliations:** National University Health System, Singapore, Not Applicable, Singapore; National University Health System, Singapore, Not Applicable, Singapore; National University Health System, Singapore, Not Applicable, Singapore; National University Health System, Singapore, Not Applicable, Singapore; National University Health System, Singapore, Not Applicable, Singapore; National University Health System, Singapore, Not Applicable, Singapore; National University Health System, Singapore, Not Applicable, Singapore; National University Health System, Singapore, Not Applicable, Singapore; National University Health System, Singapore, Not Applicable, Singapore

## Abstract

**Background:**

In the early months of the COVID-19 pandemic, the vast majority of infected persons were migrant workers living in dormitories who were young and with few medical co-morbidities. In 2021, this shifted to the more vulnerable and elderly population within the local community. We examined trends amongst the hospitalised cases, in order to demonstrate changes in disease severity in association with the evolving demographics.

Demographic shifts in hospitalised patients with COVID-19.

**Figure:**
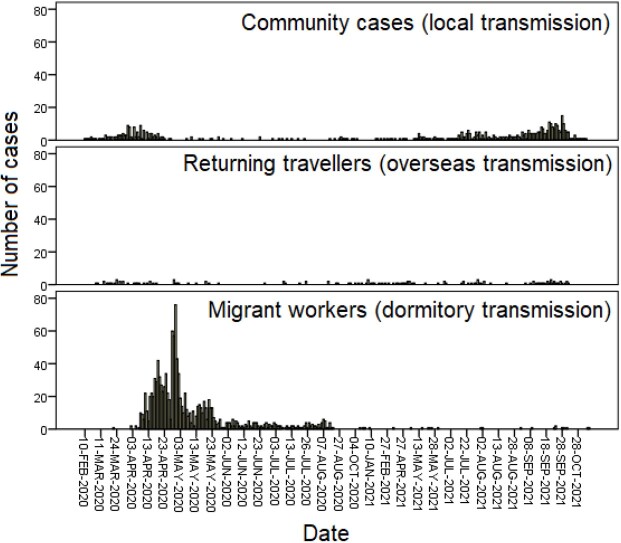

Proportion of hospitalised patients with COVID-19 requiring intensive care over time in Singapore

**Figure:**
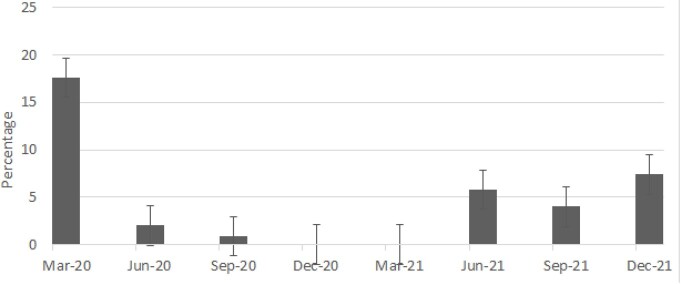

**Methods:**

All patients with PCR-positive SARS-CoV-2 admitted from February 2020 to October 2021 were included, and subsequently stratified by their year of admission (2020 or 2021). Demographics were also classified by sex, ethnicity, as well as mode of transmission, namely i) imported cases, ii) locally-transmitted cases outside of migrant worker dormitories, and iii) migrant worker dormitory cases. We compared the baseline clinical characteristics, clinical presentation and outcomes.

**Results:**

A majority of cases were seen in 2020 (n=1359), compared with 2021 (n=422), due to the large outbreaks in migrant worker dormitories. Nevertheless, the greater proportion of locally-transmitted cases outside of dormitories in 2021 (78.7% vs 12.3%) compared with 2020 meant a significantly older population with more medical co-morbidities were exposed to COVID-19. This led to an observably higher proportion of patients with severe disease, presenting with raised inflammatory markers, need for therapeutics, supplemental oxygenation and higher mortality.

Baseline characteristics of hospitalised patients with COVID-19 in Singapore over time.

**Figure:**
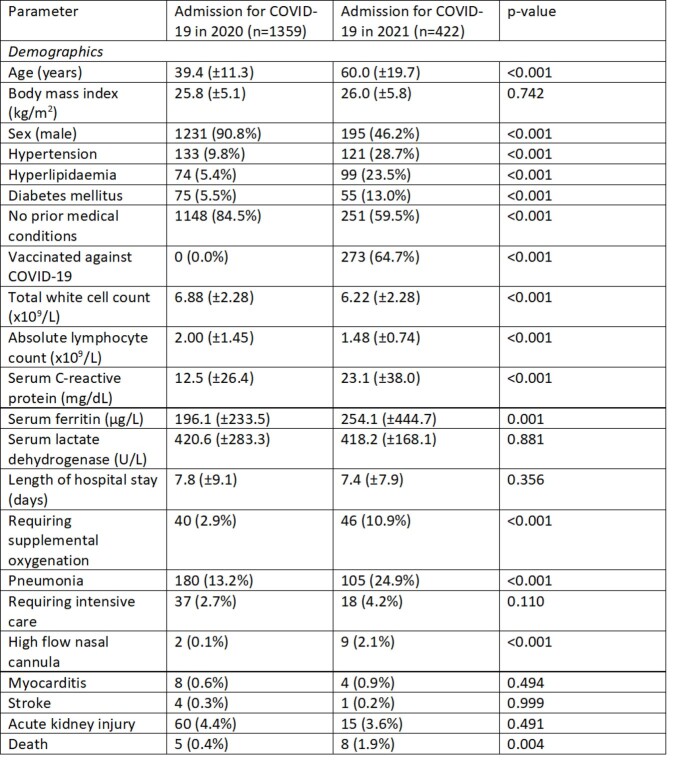

**Conclusion:**

Changing demographics and the characteristics of the exposed populations are associated with distinct differences in clinical presentation and outcomes. Understanding demographic shifts may be crucial in appropriate allocation of healthcare resources in managing hospitalised patients with COVID-19.

**Disclosures:**

**All Authors**: No reported disclosures.

